# An assessment of opportunities and challenges for public sector involvement in the maternal health voucher program in Uganda

**DOI:** 10.1186/1478-4505-11-38

**Published:** 2013-10-18

**Authors:** Jerry Okal, Lucy Kanya, Francis Obare, Rebecca Njuki, Timothy Abuya, Teresah Bange, Charlotte Warren, Ian Askew, Ben Bellows

**Affiliations:** 1 Population Council, Ralph Bunche Road, P.O. Box 17643, Nairobi 00500, Kenya; 2 Independent Consultant, Box 2753, Nairobi 00100, Kenya

**Keywords:** Challenges, Maternal health, Opportunities, Output-based approach, Public sector engagement, Reproductive health, Vouchers

## Abstract

**Background:**

Continued inequities in coverage, low quality of care, and high out-of-pocket expenses for health services threaten attainment of Millennium Development Goals 4 and 5 in many sub-Saharan African countries. Existing health systems largely rely on input-based supply mechanisms that have a poor track record meeting the reproductive health needs of low-income and underserved segments of national populations. As a result, there is increased interest in and experimentation with results-based mechanisms like supply-side performance incentives to providers and demand-side vouchers that place purchasing power in the hands of low-income consumers to improve uptake of facility services and reduce the burden of out-of-pocket expenditures. This paper describes a reproductive health voucher program that contracts private facilities in Uganda and explores the policy and implementation issues associated with expansion of the program to include public sector facilities.

**Methods:**

Data presented here describes the results of interviews of six district health officers and four health facility managers purposefully selected from seven districts with the voucher program in southwestern Uganda. Interviews were transcribed and organized thematically, barriers to seeking RH care were identified, and how to address the barriers in a context where voucher coverage is incomplete as well as opportunities and challenges for expanding the program by involving public sector facilities were investigated.

**Results:**

The findings show that access to sexual and reproductive health services in southwestern Uganda is constrained by both facility and individual level factors which can be addressed by inclusion of the public facilities in the program. This will widen the geographical reach of facilities for potential clients, effectively addressing distance related barriers to access of health care services. Further, intensifying ongoing health education, continuous monitoring and evaluation, and integrating the voucher program with other services is likely to address some of the barriers. The public sector facilities were also seen as being well positioned to provide voucher services because of their countrywide reach, enhanced infrastructure, and referral networks. The voucher program also has the potential to address public sector constraints such as understaffing and supply shortages.

**Conclusions:**

Accrediting public facilities has the potential to increase voucher program coverage by reaching a wider pool of poor mothers, shortening distance to service, strengthening linkages between public and private sectors through public-private partnerships and referral systems as well as ensuring the awareness and buy-in of policy makers, which is crucial for mobilization of resources to support the sustainability of the programs. Specifically, identifying policy champions and consulting with key policy sectors is key to the successful inclusion of the public sector into the voucher program.

## Background

The persistent poor maternal and child health outcomes in sub-Saharan Africa raise questions as to whether the Millennium Development Goals 4 and 5 of reducing child and maternal mortality ratios by two-thirds and three-quarters, respectively, between 1990 and 2015 will be realized in many African countries [1-3]. Many countries in the region not only score poorly on maternal and reproductive health (RH) indicators but also face wide poor-rich inequalities, with the poor bearing a disproportionate disease and economic burden [4-6]. Ensuring access to maternity services such as antenatal care, attended skilled deliveries at facility, and postnatal care can result in better maternal and infant health outcomes and help to attain the Millennium Development Goals [7-9].

In Uganda, maternal mortality appears to have declined slightly over the past decade from 505 deaths per 100,000 live births in 2000/2001 to 438 deaths per 100,000 live births in 2011 [10,11]. However, maternal mortality would have to fall to 131 deaths per 100,000 live births by 2015 to meet the Millennium Development Goal for maternal health [4,10,11]. The proportion of pregnant women seeking at least one antenatal care visit is notably high at 95%, while 57% of women deliver in a health centre or hospital, meaning that more than a third of pregnant women make contact with the health system during pregnancy but do not return to give birth. There are also wide regional and economic disparities in deliveries assisted by skilled attendants; 94% of deliveries in Kampala are assisted by skilled attendants as opposed to only 42% in the southwestern region [11]. Among the poorest quintile, the proportion of births attended by skilled health personnel was 29% compared to 77% among the wealthiest 20% [12,13].

To address limitations of current healthcare systems and tackle financial barriers to access, alternative healthcare financing approaches have been developed that link pro-poor provider payments to outputs. One strategy – referred to as the output-based approach (OBA) – targets priority health services to underserved and/or marginalized populations and reimburses approved health facilities for seeing beneficiaries on a clearly defined service package [14-16]. OBA RH voucher programs have been designed to increase access to sexual and RH services for new users who, in the absence of the voucher, would probably not have sought care [16]. Available evidence suggests that voucher programs can increase utilization and efficiency of health services and improve quality of services and health status among target population groups [16-18]. In both Uganda and Nicaragua, voucher programs were associated with decreases in prevalence of selected STIs [19,20]. In Bangladesh and Pakistan, voucher programs were found to increase equity in access to healthcare among the poor [21,22]. Similar RH voucher programs have since been implemented in Kenya, Uganda and Tanzania with varying success. The Kenya and Tanzania voucher programs are anchored within the public health system, while the Uganda program currently operates only in private sector facilities.

The maternal health voucher program in Uganda was launched in late 2008 with three years of funding from the German Development Bank and the Global Partnership on Output-Based Aid (GPOBA-World Bank). The program was implemented in 20 districts in southwestern Uganda. Low-income women who qualified could purchase the voucher for 3,000 Uganda shillings (approximately US $1.40) to access safe motherhood services (four antenatal care visits, delivery, and postnatal care up to six weeks). Community-based voucher distributors are responsible for targeting poor pregnant women using a regional-specific poverty grading tool. The voucher program is being implemented by Marie Stopes International-Uganda, which acts as the voucher management agency on behalf of the Ministry of Health (MoH)-Uganda [20]. Currently the program accredits private for-profit and not-for-profit facilities that meet accreditation standards for basic or comprehensive emergency obstetric care. Public sector facilities have limited engagement as referral points, without any voucher reimbursement. Public facilities were initially not contracted due to concerns that funds disbursements and management would be pooled at the district level and that public facility administrators lacked sufficient autonomy to effectively use the OBA reimbursements. Some district and national level officials also viewed introducing the voucher program in public health facilities, with vouchers nominally sold to poor clients, as in conflict with the government policy of providing services at no cost.

Evaluation results of the Uganda voucher scheme show some measure of success. From September 2008 to June 2011 a total of 104,547 maternal health (*HealthyBaby*) vouchers were sold, of which 56% were redeemed for delivery services while 81% were redeemed for at least one antenatal care visit. In a quasi-experimental evaluation, the Uganda voucher scheme was associated with increased use of safe motherhood services, improved knowledge of danger signs during pregnancy/childbirth, and reduced inequity in the utilization of safe motherhood services between poor and non-poor women. In contrast, the results showed that births delivered in public health facilities in the study area fell over the two year period [20]. In a separate analysis, voucher sales and claims data showed a positive correlation between the number of contracted facilities in a district and the number of vouchers redeemed for delivery services as well as the proportion of births to poor women attributable to the program [23]. This, not surprisingly, suggests that access and proximity to facilities are important factors influencing the uptake of voucher services.

Within the context of these results, use of health services can be understood as directly influenced by both demand-side determinants (often the ability to use health services at individual, household or community level) as well as by supply-side determinants (mostly aspects inherent to the health system that hinder service uptake by individuals, households or the community) [24]. Thus, it is imperative that demand-side and supply-side barriers be addressed in tandem to increase equity in use of RH services [21,24,25].

Engaging the public sector in the Uganda voucher program is an important mechanism to address inequities in coverage and to improve the long-term sustainability of the initiative. It would also give the government a greater stake and increase the likelihood that government, at both national and district level, is more engaged in the program. Involving public sector in the service delivery would also ensure that more women will benefit from quality RH services. In this paper, we explore the potential for inclusion of public sector health facilities in the voucher program. As with other public health-related issues, an understanding of health manager’s perspectives can improve understanding and help identify policies, structures, networks, and individuals with whom to link in order to ensure successful implementation.

## Methods

This study used a qualitative approach to identify district and facility administrators’ understanding of the challenges to seeking care and how the voucher program can address the constraints, as well as opportunities and challenges for public sector involvement in the voucher program. A purposeful sample of six district health officers and four public hospital medical superintendents within the voucher program districts were selected in consultation with the voucher management agency. All the informants had worked in their current posting for a minimum of two years at the time of the interviews. An open-ended key informant interview guide addressed three broad areas: barriers to seeking RH services, identify the constraints and opportunities for public sector involvement in the voucher program, and suggestions for addressing the challenges. One research assistant familiar with the Uganda voucher program and the beneficiary communities was trained to conduct the interviews.

Following introductions and explanation of the study, written informed consent was obtained from each of the informants and interviews were conducted in English. All interviews were tape-recorded and transcribed verbatim. Transcripts were coded using QSR NVivo 10 Software and analysed using content and thematic analysis [26,27]. Content analysis approach was adopted because it suits the analysis of this type of qualitative data. Transcripts were read and reread by two researchers to identify recurrent themes and develop a coding tree. After coding, memos were developed to examine each code for sub-themes, nuances, apparent contradictions, and patterns across the interviews. Key themes emerging were further placed within a thematic framework which were sorted hierarchically into main and sub-themes.

## Results

We identified five key themes from our analysis; however, in this paper, we focus on four themes that expand the understanding of public health sector engagement in the Ugandan RH voucher program. We placed emphasis on barriers to utilization of RH services and how to address the barriers, as well as opportunities and challenges for involving government facilities in the program. In many cases, the informants also referred to their knowledge of the existing voucher program within their local network and reflected on their working relationships with staff in voucher-accredited facilities.

### Barriers to utilization of reproductive health (RH) services

Several cross cutting issues emerged from the interviews when informants were asked about the prevailing trends and the most pressing RH issues in their specific regions. Key RH issues identified included the low utilization of family planning, antenatal, and facility delivery and postnatal care services. While approximately 90% of most mothers attended at least one antenatal care visit during pregnancy, all the informants noted the low utilization of facility delivery services, estimated at below 50%, low contraceptive prevalence rate estimated at 23%, and STI services estimated at 30%.

One of the key themes that emerged dealt with supply-side barriers that negatively affected accessing RH services in both public health facilities and private facilities. Most interviewees mentioned that public health facilities are understaffed with too few doctors and midwives to attend to the high volume of clients. The problem of understaffing increases workload and risks provider “burn-out” leading to poor RH service provision. Informants noted that understaffing in facilities often led to unnecessary delays in service provision, poor record keeping and under-reporting of essential maternal, child, and other health indicators.

*“But now the health sector … as a result of under staffing and poor working conditions both at the place of work, and poor welfare; for example our antenatal here you can get up to 100 mothers a day and there are only two or three midwives to see these mothers so quality can go down.”* Hospital Medical Superintendent

In addition to being overworked, service providers were said to be poorly remunerated leading to low morale often translating to negative provider attitudes. Poor provider attitudes were linked with low utilization of RH services. Similarly, many public health service providers were said to be running parallel activities leaving them with little time to attend to clients.

Interviewees also felt that lack of supplies and essential commodities for RH services led to low uptake of RH services. The perennial lack of drugs and non-pharmaceutical supplies necessary for RH services (e.g., vasectomy kits, bilateral tubal ligation kits, delivery beds and delivery sets) means that potential clients may be reluctant to seek services, especially if this entails covering long distances in bad terrain with the possibility of not getting the required service. The narratives showed that a vast majority of the population live in the rural areas where there are few facilities facing supply and staff shortages.

On the other hand, demand-side factors were also said to contribute to low uptake of RH services in the region. Eminent among these are socio-economic and cultural factors such as lack of knowledge, misconceptions, male involvement, and poverty. For instance, informants alluded that most women discreetly accessed some RH services such as family planning due to perceived resistance from their partner’s rendering long-term use of such services unsustainable. Similarly, the overall lack of male involvement around RH issues complicated early decision making for delivery services. In particular, informants reported low utilization of services in cases where partners were not fully involved or did not support decision making around accessing RH services. This situation is even dire where the woman is entirely financially dependent on the man. In such cases, there was a near lack of utilization of RH services if the man is not supportive.

More so, utilization of RH services was reported to be low where there were financial costs associated with the service. For example, most women opted to deliver at home because most public health facilities require them to bring along personal supplies like bedding, clothing and basins, which means they have to incur some costs on the items as well as travel costs in cases where they live far away from the health facility.

Socio-cultural factors also contributed to the low utilization of facility delivery services in the region. In the target community, traditionally, pregnant women travel to their ancestral homes just before childbirth to get support and care from their close relatives. This cultural practice means that most often than not, women end up being supported through delivery by their close relatives or traditional birth attendants who are in close proximity to the women and are equally viewed as experienced in attending to childbirth.

*“For example a person may be working here in Hoima but her home area is in Kasese so when she is about to deliver, she has attended antenatal here, she travels to Kasese in the village where her mother will look after her and in the process she is moving away from where the service is to where there is no service.”* District Health Officer

Self-efficacy or normalization of childbirth was also cited as a major barrier in accessing facility delivery services. This is illustrated in the quote below from one of the informants.

*“…Self efficacy in that delivery is viewed as normal and I’ve done it before why bother to go to the doctor with a voucher card.”* District Health Officer

### Suggestions on how to address the current barriers to utilization of RH services under the voucher program

With relatively low utilization of RH services even among voucher beneficiaries, informants were asked for suggestions on how to improve uptake of services under the voucher program. Suggestions fronted included intensifying ongoing health education in the communities through sensitization targeting men and women, community focal persons and opinion leaders. The use of varied media channels like print and audio was also mentioned. Specifically, informants also suggested the involvement of community focal persons especially the village health team (VHT) in the program.

*“Involve the VHT. VHTs have done a lot. Actually after their training, there is a lot they do to mobilize the community. Those few of them are still there; but the outcome can become better when the VHTs get involved in the communities; so that we get linked to them on phone and they do continuous mobilization.”* District Health Officer

Also reported was the need to integrate the voucher program with other existing services to enhance uptake of RH services. Undertaking this, especially within the existing public health sector, was seen as critical as this would ensure that communities are mobilized using existing structures and that quality health care services are offered to all clients.

Another aspect reported was the need to provide transport under the voucher system. This was viewed as important among rural communities where poverty levels are high and the distance to the nearest facility is long. Thus, informants felt that providing transport or even having outreach services for essential RH services would open up access to important services such as family planning and antenatal care. Potentially, according to interviewees, the transport problem could be addressed by using motor bike riders also known locally as *boda boda* to transport women in need to the health facility. Generally, *boda boda’s* are cheap and are literally common in most rural and urban settings in Uganda.

*“So meaning that if we got a boda boda, every village somehow currently has a boda boda. If a boda boda guy, uhh knows that if I go to a health facility, if I keep ferrying mothers to a health facility, I will get a transport refund at the end of the month. Simple, it all changes the picture so the way this works, is the transport voucher is linked to the facility….”* District Health Officer

Informants also felt that more facilities should be accredited, including public facilities, to increase geographical coverage and thereby taking services closer to the people. Public facilities have a wider geographical spread when compared to private facilities currently accredited in the program. Further, informants felt that the voucher program needed to expand in coverage to reach more districts in the region as the entire southwestern region was regarded as being generally poor.

In addition, informants reported the need for continuous monitoring and evaluation of the voucher program to assess challenges and ways of addressing them. This, they intimated, would provide an in-depth understanding of the operations of the program and help identify solutions for addressing barriers to utilization of services.

*“The advice I can give you is, if you had started it, try to follow it up try to continue studying it to see where it is not functioning or where there are problems; address them immediately; have continuous communication with the facilities with whom you’re working with.”* District Health Officer

There was also consensus among interviewees that the voucher program would improve staff morale through a better working environment where supplies were steady and, combined with the output orientation, leading to improved client-provider interaction. In addition, interviewees felt that, by being involved in the voucher program, health facilities would likely generate more money and use the funds to expand the facilities and recruit more staff to cater for the additional demand. They felt that, as a consequence, many voucher users would access services and benefit from quality RH services.

*“Extra funding from the voucher program can be used to hire staff, improve pay, purchase supplies and improve general quality of services. With an improved work environment, staff morale is also likely to go up. Enhancing capacity building will also help to improve staff morale.”* Hospital Medical Superintendent

Finally, informants felt that introduction of the voucher program in the public sector could help address some of constraints to utilization of RH services. They were of the view that the program’s success in private for-profit and not-for-profit facilities could well apply in public facilities.

*“The voucher program has facilitated these facilities (those in the voucher program) in terms of some kind of payment. The staff involved were motivated and they were able to work well. Mothers were actually pleased with this.”* Hospital Medical Superintendent

### Opportunities for public sector involvement in the voucher scheme

Although there is minimal engagement between the voucher program and public sector at present, during discussions, interviewees drew on their knowledge of the existing voucher program and their relationship with their local networks to reflect on the opportunities for the public sector involvement in the voucher scheme. Informants saw numerous advantages for engaging the public sector in the voucher scheme. Primarily, the public sector was seen as being well positioned to provide voucher services by virtue of having country wide reach and the necessary health infrastructure. The specific points discussed are presented below.

#### ***Geographical spread***

Interviewees recognized that contracting public facilities was likely to increase RH service delivery and coverage as public facilities are geographically widespread across the region. This, it was also felt, would also help district level coordination of RH service delivery, decongest current voucher facilities in high population areas, encourage more needy women to seek care and increase demand for RH services in rural areas.

Informants felt that the location of some accredited facilities in towns or trading centres put rural voucher clients at a disadvantage. This, coupled with the poor state of the roads and bad terrain, were cited as major hindrances to timely access of medical care especially in emergency situations.

*“… So if the government facilities, because most of them are rural, if they are also facilitated in offering or receiving voucher clients, then these clients would actually be at those points, and the cost of transport cut down as they can actually walk to the health facility.”* District Health Officer

Some informants identified a strong link between transport problems and the low uptake of safe motherhood services. In the opinion of most interviewees, long distances to the accredited facilities and lack of transport money likely explains the current low uptake of voucher services. As indicated above, some accredited facilities are located in distant location in towns or trading centres which put rural voucher beneficiaries at a disadvantage. To address this situation, some informants suggested that the number of voucher-accredited facilities be increased, with special attention given to public facilities in rural areas and expanding mobile health units in remote areas. In addition, others also suggested the introduction of transport vouchers which could cushion the poor from high transport costs and motivate them to access health services.

*“First of all the services; the services are not readily available, because access is very limited as a result of the population being rural where we have only few facilities that are grossly understaffed.”* District Health Officer

*“…one way to do it is to provide a transport voucher in addition to the other (health-based) voucher for the government [facilities].”* District Health Officer

#### ***Enhanced services and referrals***

The informants generally agreed that most public health facilities are well equipped to handle obstetric complications. Contracting public facilities would build from the current practice among voucher accredited facilities to regularly refer patients with complications to larger public facilities. Under the existing arrangement, referred clients are registered at no fee and treated like any other client, with all the attendant resource challenges in the public sector. Contracting public facilities would potentially bring additional revenue for a service that currently may not be well financed.

*“Every week, we get referrals… you see in the private sector, they are not allowed to transfuse blood if a mother needs blood they just refer to a hospital… if the mother cannot deliver normally in the private facility they are also referred for operation.”* Hospital Medical Superintendent

It was felt that the large infrastructure and range of programs available in regional and larger district hospitals could be better utilized in the voucher program. In particular, it was noted that community outreach and sensitization activities, which are coordinated through district and regional hospitals, could act as channels for sensitizing communities and offering voucher-related health education activities.

*“…well first of all you have to sell the program. Spread to the people during antenatal visits the benefits (of the voucher program), give them the possible challenges, the difficulties associated with uuhh seeking services … among the informal sector hmmm traditional healers, hhhmmmm delivering themselves. You know actually it’s posing to them, the possible risks that are associated with home deliveries.”* District Health Officer

#### ***Contracting voucher services in private wings at public facilities***

There were also proposals from the interviewees that the voucher program can operate within existing or yet-to-be established private wings of public facilities to circumvent any operational issues, especially with respect to the government policy of providing services at no cost in the public sector. In Uganda, user fees were abolished in all government health facilities in 2001; however, a dual system was later established in some health facilities: one health facility wing offers services at no cost for those who cannot afford payments while another wing charges user fees for those who can afford to pay [28,29]. In view of this, informants saw the potential to contract services from the private wing for voucher clients.

*“We are going to get a private wing and that one will work… let’s imagine that it is just general services that will become very difficult. It will work with the private wing.”* Hospital Medical Superintendent

*“… if you can put it in the private wings then the voucher program could operate there.”* District Health Officer

Informants strongly believed that the current government policy of providing services at no cost may be a barrier in implementing the voucher program within the public sector. They felt that charging clients a nominal fee for the voucher to then access services in the “no fee” public system could be viewed as creating a parallel program countering the government policy of offering “free health services to all”. In view of this situation, some interviewees proposed introducing the voucher program in the government private wings as these entities are allowed to charge some fees.

*“ In government health facilities, particularly, I see it as a problem because in government health facilities everything is supposed to be free so I don’t know how they can buy the voucher, for example now what I heard, they were buying the voucher at UGSh3000”* District Health Officer

The argument of introducing the voucher program in government private wings was cautionary and made in the context of the uncertain political class reaction to the introduction of the voucher program. Most interviewees felt that given the sensitivity of health care in Uganda, a section of the political class could hijack the process and claim that the government is reneging on its promises to provide “free” health care to all by charging service costs.

Other than these concerns, interviewees generally felt that introduction of the voucher program in the public sector could help address some of the RH accessibility constraints. They were of the view that the program’s success in private for-profit and not-for-profit facilities could well apply in public facilities.

### Challenges contracting public facilities

Perceived challenges for public sector engagement in the voucher program included the government policy of providing health services at no cost, the challenge of sensitization in many communities, and the risk of political opposition as well as the potential for low morale among healthcare workers not linked to the program.

Interviewees observed that introducing the voucher program in public health facilities would require proper planning, community sensitization, and accountability to ensure a smooth implementation process and transparent funds management. They noted that the introduction of the voucher program in the public health facilities would most likely be enhanced with community sensitization and RH awareness campaigns as well as through gaining local political support from the national and district health offices. It was also mentioned that gaining political support is a critical prerequisite for successful engagement of public facilities in the RH voucher program. Some of the interviewees felt that buy-in from the political class was a necessary step to counter any negative stance that the public health facilities may take on the voucher program.

*“… in the public sector we have a problem because services in public sector … are supposed to be free and provision of services is the sole responsibility of government to provide those services and I would find it very hard for the voucher system to work in public because in the public we do not receive cash from clients and of course if they received cash from the project that means there have to be issues of planning, accountability and all that and of course that will also distort other services.”* District Health Officer

At the same time, there was the emergent concern from participants that if the voucher revenues were to be shared among health workers as salary top-ups, that it could negatively influence the motivation of health providers not linked to voucher services. Participants also observed that introducing the voucher program in specific departments like RH could affect service provision in other departments. As such, it was felt that staff members who are not involved in the program may feel excluded and de-motivated to work in their respective departments. Similarly, since voucher payment is based on output, interviewees were concerned that service providers could potentially concentrate on voucher clients at the expense of non-voucher clients in their department. According to the interviewees, possible solutions to this problem would be to expand the voucher benefit package to cover other non-RH services, involve additional staff, and ensure that incentives could be shared with non-voucher staff.

### Mechanisms for addressing operational challenges

Participants were also asked potential mechanisms to overcome operational challenges and ensure efficient service delivery. Participants proposed opening dialogue with stakeholders, alternative funding routes for public facilities, sensitizing the community and political class, using revenue from the voucher program to improve facilities such as hiring of staff and purchasing supplies and equipment as ways of addressing these challenges. Table 1 summarizes the operational challenges and suggestions for addressing them.

**Table 1:** Operational challenges of introducing voucher program in the public sector and ways of addressing them

**Operational challenges**	**Mechanisms to address the challenges**
There exists a lot of bureaucracy at the senior government levels which can delay the process of engaging the public facilities in the voucher program and later managing the relationship between VMA and public facilities.	*▪ “There should be dialogue from the national level between the MoH and VMA to come to a consensus on how to introduce the program to the public sector without contradicting government policy.”*
Public facilities are traditionally run by government funds at the district level. Will voucher funds go to district office or will they go directly to the facilities?	*▪ “Funding should come in directly to the facilities to cut down the burden of bureaucracy and prevent [potential] fraud.”*
	*▪ “A memorandum of understanding can be signed between the VMA and each hospital management to address the concerns.”*
How will the voucher program operate in line with government policy of providing free health for all?	*▪ “Voucher program can be introduced on the back of government sanctioned private wards where clients are already paying for services.”*
	*▪ “Politicians, religious leaders and other community gate keepers should be involved so that the program can gain acceptability within the communities.”*
Would under-staffing of public hospitals and lack of RH supplies negatively affect quality of OBA services? How will public facilities benefit from funds accrued from the voucher program?	*▪ “Extra funding from the voucher program can be used to hire staff, improve pay, purchase supplies and improve general quality of services. With an improved work environment, staff morale is also likely to go up. Enhancing capacity building will also help to improve staff morale.”*
What is the sustainability of the RH voucher program after the end of OBA program/funding?	*▪ “Private wards operate in many public referral hospitals and the voucher program could be contracted to operate there. With funding from the voucher program, the private wing will be able to build facility capacity. Depending on the duration of the program, the infrastructure investment could be used beyond the life of the program. Better services in these private wings will encourage utilization for non-voucher, paying clients and will help to sustain the facility.”*

MoH: Ministry of Health; VMA: Voucher Management Agency; Output-based Approach.

## Discussion

Like many health systems in the region, the Uganda health care system is hampered by several challenges which contribute to the low uptake of facility delivery services. Public health facilities, where a policy of free health care services is in force, are faced with perennial shortages of essential medical supplies. Approximately 72% of government health units have monthly stock outs of medicine and supplies and are often poorly staffed [30]. In terms of health care expenditure in Uganda, households in aggregate contributed 49% of the national health expenditure out-of-pocket, the central government contributed 15%, while development partners and international non-governmental organizations combined contributed 36% in the 2009/2010 financial year [31]. Other studies have identified the perceived low quality of care in facilities, distance to health facilities, transportation challenges, costs of services including informal charges or expenses, provider attitudes, power dynamics including ineffective decision making at the household level, and socio-cultural norms as some of the factors that affect the uptake of services in the country as well as in similar settings [32-41]. Faced with these challenges, the effective decision of most poor mothers is not to access facility delivery services.

Findings from this study provide useful insights on barriers to utilization of RH services and how to address the barriers under the voucher scheme as well as opportunities and constraints of engaging the public sector facilities in the maternal health voucher program in Uganda. The analysis shows that supply-side and demand-side issues at facility and individual level as well as operational and health policy barriers affect utilization of RH services and are common factors affecting service delivery at public health facilities in sub-Saharan Africa [13,24].

Although challenges with health service uptake exist, our findings highlight the advantages of engaging the public sector in the voucher program in Uganda. These include the available large infrastructure and wide range of services and referral networks in the public sector. For example, the large infrastructure and networks of public health facilities can improve client referrals, community sensitization, and education on specific RH issues. Public sector strategies like the Presidential Initiative on AIDS Strategy for Communication to Youth and the use of the VHTs which have shown success in other health programs in Uganda can be replicated in the voucher program to improve access to quality RH services, especially among rural women [42,43].

In relation to engaging the public sector in the voucher program in Uganda, our findings can be used as a basis for thinking through the process of practical implementation, trouble-shooting and policy shifting. This can be done through framing the research findings in ways that resonate with existing health policies. Similarly, communicating research evidence should be done in a way that strengthens networks and relationships with policy networks, policy “champions”, and consultations with key policy actors. Nutley et al. suggest that the role of leadership and support in research communications is inevitable on promoting evidence-based practice [44]. In the Ugandan context, findings from this study could potentially be used to steer ongoing discussions and planning activities between World Bank and the MoH for a national scale-up of the RH voucher program in Uganda.

Despite the opportunities for introducing the voucher program in public sector facilities, we found that health policy and operational issues are key barriers to engaging public facilities in the voucher program. Technically, these barriers can be separated into three key aspects. The first being the government health policy of offering “health services for all”, which mandates the state to provide “free” health services for all and requires effective alternative facility revenue options for successful implementation. As in the case of many countries in the region, provision of “free” health services is more a theory than a reality [13,45]. In practical terms, the policy of offering nominally free health services could have implications for the implementation of the voucher program in public facilities. Potential clients may doubt that services are really available at no additional cost beyond the nominal voucher fee. At the same time, there are uncertainties at how politicians would react upon the introduction of the voucher program in public sector facilities which supposedly provide “free” health services. As such, informants felt that anchoring the voucher program in the private wings of public hospitals would avert any opposition to the voucher program since private wings in public hospitals have been in existence in Uganda for a long time. The second key aspect of the public sector engagement in the RH program is the ability to address the operational issues such as government bureaucracy, disbursement of funds, supplies and equipment, staffing, and quality assurance, so as to ensure smooth operations of services and optimizing resources. The third element, and perhaps the most important for addressing the challenges, is the implementation of a strict monitoring and oversight system, which routinely and systematically reports quality assurance issues, availability of supplies, and personnel productivity, thereby permitting evaluation of services in the public facilities and responding as the need arises.

Whereas the challenges in implementing vouchers in the public sector are significant, evidence from the Kenya voucher program shows that it is possible to involve both the public and private sectors in the voucher program [15,46]. The introduction of the voucher program in public health facilities in Kenya was made possible by building relationships and engaging policy “champions” and consulting with key policy sectors. The successful implementation of the voucher program in the public sector requires strong leadership at national, district, and community levels. The leadership buy-in at various levels is crucial for mobilization of resources to support the sustainability of the programs [46]. Moreover, the involvement of both public and private facilities in the program not only widens client choice thereby improving service uptake but also strengthens linkages between the two sectors through public-private partnerships and referral systems.

It is, however, worth noting that this study has some limitations. Although the selection of participants allowed us to capture a range of perceptions and experiences, the sample is small, covering only six districts in southwestern Uganda where the current voucher program is operational and may not be representative of the situation in other parts of Uganda. Similarly, we did not collect information on user’s (clients) perspectives which would have strengthened the study findings. As such, the emergent themes are specific to the provider’s views on RH and the voucher program and caution is needed before broadly generalizing these findings beyond the context from which they were drawn.

## Conclusions

This study identifies contextual, policy and health system challenges that the voucher program in southwestern Uganda is likely to face if it is to effectively engage with the public health sector. These challenges can be overcome through proper planning as well as sensitization and engagement with community members, facility managers, healthcare workers, and policy makers. The involvement of the public sector in the program would, in turn, help decongest facilities in high population areas, reach out to more needy women and increase demand for and use of priority RH services while channelling additional resources to under-financed public facilities.

## Abbreviations

OBA: Output-based approach; RH: Reproductive health; VHT: Village health team.

## Competing interests

The authors declare that they have no competing interests.

## Authors’ contributions

JO involved in data analysis, interpretation, drafting, organizing and overall revision of the manuscript. LK involved in data collection, interpretation of the data, re-organizing and revision of the manuscript. FO involved in interpretation and the revision of the manuscript. RN involved in the revision of the manuscript. TA involved in the revision of the manuscript. TB involved in data analysis and revision of the manuscript. CW involved in the revision of the manuscript. IA involved in the revision of the manuscript. BB involved in conceptualizing the study and revising the manuscript for intellectual content. All authors read and approved the final manuscript.
